# Measuring Regional Performance in the Italian NHS: Are Disparities Decreasing?

**DOI:** 10.1007/s11205-021-02775-8

**Published:** 2021-08-30

**Authors:** Silvia Bruzzi, Enrico Ivaldi, Marta Santagata

**Affiliations:** 1 Department of Economics, University of Genoa, Genoa, Italy; 2 Department of Political Sciences, University of Genoa, Piazza Emanuele Brignole 3a canc, Genoa, Italy; 3 The Italian Centre of Excellence on Logistics, Transport and Infrastructures, C.I.E.L.I., Genoa, Italy; 4 Centro de Investigaciones en Econometría, CIE University of Buenos Aires, Buenos Aires, Argentina

**Keywords:** Health Care, Health Status, Life Style, Regionalisation, Composite Index, Italy

## Abstract

Given the regional disparities that historically characterize the Italian context, in this paper we propose a framework to evaluate the regional health care systems’ performance in order to contribute to the debate on the relationship between decentralisation of health care and equity. To investigate the regional health systems performance, we refer to the OECD Health Care Quality Indicators project to construct of a set of five composite indexes. The composite indexes are built on the basis of the non-compensatory Adjusted Mazziotta-Pareto Index, that allows comparability of the data across units and over time. We propose three indexes of health system performance, namely Quality Index, Accessibility Index and Cost-Expenditure Index, along with a Health Status Index and a Lifestyles Index. Our framework highlights that regional disparities still persist. Consistently with the evidence at the institutional level, there are regions, particularly in Southern Italy, which record lower levels of performance with high levels of expenditure. Continuous research is needed to provide policy makers with appropriate data and tools to build a cohesive health care system for the benefit of the whole population. Even if future research is needed to integrate our framework with new indicators for the calculation of the indexes and with the identification of new indexes, the study shows that a scientific reflection on decentralisation of health systems is necessary in order to reduce inequalities.

## Introduction

Health care in Italy has been the subject of an important regionalisation process started in 1992, that anticipated the constitutional reform by about ten years.

The 1992 reform assigned to the Regions the responsibility of both organising the provision of health services and guaranteeing the economic sustainability of the health system at the regional level (Mossialos & Maynard, 1999).

This reform was based on principles of decentralization consistent with vertical subsidiarity, which was going to be enhanced in the process of European integration in those years (Müller-Graff, 1996), and with a general downsizing of the role of the State, common to all European countries (Velo, 1993).

The main risk identified by the debate at that time was that regionalisation in a country like Italy, historically characterized by a strong North–South economic and social dualism that persisted in the 1990s, could favour the development of very different regional systems in terms of guaranteed services and economic performance, in contrast with the Italian Constitution (Bruzzi, 1996).

The academic debate shows that this issue is still open today, as there are several papers (e.g. Di Novi et al., 2015, 2019; Nuzzo et al., 2018; Toth, 2014) that use different approaches to investigate the relationship between decentralization and equity in health care in Italy.

Within this framework, this paper aims to contribute to the evaluation of the regional health care systems’ performance with the specific purpose of investigating whether the regionalisation process is overcoming regional imbalances.

The issue of performance measurement in health care has also been addressed at international level with the aim of promoting an increasing focus on continuous improvement of health care systems. International institutions, like the World Health Organization (WHO) and the Organisation for Economic Co-operation and Development (OECD), had and still have a central role in encouraging performance measurement at the national level. The OECD, given the proliferation of health indicators and the need to build conceptual frameworks, started the Health Quality Indicator (HCQI) project in 2004, with the aim of developing a set of indicators to investigate the quality of health performance at national levels. Since the OECD mission is the promotion of policies that improve the economic and social well-being of all citizens in the light of the disparities which persist at the national level, it seems necessary to maintain a high level of attention to the national experiences.

In this process, at the European level, a particularly important step is the Tallin Charter, which provides a strategic framework to strengthen the health system in the WHO European Region. Adopted by all members of the European Union during the European session of the WHO Regional Committee, in September 2008 (resolution EUR/RC58/R4), the charter has the aim of giving the health system a prominent place on the political agenda of the Member States. One of the central messages of the Charter is that a well-functioning health system, both in terms of health and efficiency, is essential for every society. To this end, Member States committed to promote transparency and accountability. This process enhanced the prominence of accurate and up-to-date information on performance in order to improve health systems. Without performance information it is impossible to identify strengths and weaknesses of a health system, and consequently it is impossible to promote its improvement (Smith et al., 2009).

With the aim of assessing the performance of regional health systems in compliance with this institutional framework, we propose a set of composite indexes constructed according to HCQI project by the OECD. More precisely, we propose three indexes of health system performance, namely *Quality Index*, *Accessibility Index* and *Cost-Expenditure Index*, along with a *Health Status Index* and a *Lifestyles Index*. To construct the composite indexes, we opt for a non-compensatory approach, i.e. the Adjusted Mazziotta-Pareto Index that allows comparability of the data across units and over time (Mazziotta & Pareto, 2018).

The paper is divided into 5 sections. Section 2 describes the institutional background by illustrating the state of the art of the reform of the Italian National Health Service (NHS) and the conceptual framework. Section 3 describes the methodology adopted and the data used for the construction of the composite indexes. Section 4 describes and discusses the results. In Sect. 5 some conclusions for future research are drawn.

## Institutional Background

### The Italian National Health Service and its Regionalisation

Health care in Italy has been the subject of important regulatory measures for several years. The National Health Service was established in 1978 with a first reform that replaced the previous mutualistic system, still unfinished at the time (Dirindin & Vineis, 2004), introducing a Beveridgean health system, on the model of the British one dated 1948 (Bruzzi, 2012; Velo & Bruzzi, 2004).

The reform aimed to guarantee universal and global health insurance coverage, also through a widespread network of local providers (Unità Sanitarie Locali, USL), able to cover all the needs of the population (prevention, hospital, rehabilitation, etc.). In those years in Italy the territorial imbalances in the socio-economic conditions of the population, as well as the gaps in the insurance coverage and in the distribution of local providers, were in fact still very strong (Bruzzi, 1996).

The National Health Service established in 1978 was further reformed in the early 1990s when, in the framework of the process of monetary integration, the control of public expenditure levels became a priority (Taroni, 1996). In particular, the reform introduced in 1992 aimed to ensure the economic sustainability of the health system and, at the same time, to provide a qualitatively and quantitatively adequate level of services for the entire Italian population (Bruzzi, 2006; Velo & Pellissero, 2002).

The reform was based on principles of decentralisation in line with vertical subsidiarity, strengthened in the process of European integration in those years, and with a broad downsizing of the role of the State, common to all European countries (Müller-Graff, 1996; Velo, 1993). If the institution of the NHS in the 1970s centralized decision-making power in the hands of the central public actor, the reform of the 1990s launched a process of regionalisation of health care that anticipated the constitutional reform by about ten years. Indeed, the reform assigned to the Regions, as institutions closer to the citizens and their needs, the responsibility of organizing the provision of health services and guaranteeing the health system economic sustainability at regional level (Mossialos & Maynard, 1999). The economic-financial responsibility of the Regions is a fundamental characteristic of this reform, since health expenditure during the construction phase of the NHS in the 1980s had proved to be out of control.

In the 1990s, the debate on the reform identified a major risk. The regionalisation of a country like Italy—historically characterized by a strong North–South economic and social dualism persisting in the 1990s—could favour the development of very different regional systems in terms of guaranteed services, in contrast with the Italian Constitution (Bruzzi, 1996).

In the early 2000s the Constitution was reformed and responsibility for the organisation of health care was assigned to the Regions, while the State, in order to mitigate the risk of inequalities, was assigned the task of defining the social insurance package (Livelli Essenziali di Assistenza, LEA)^1^ that the Regions must guarantee to all Italian citizens.

In the new model of governance of the Italian health care system, decisions concerning the funding of the system are taken within the State-Regions Conference through the so-called State-Regions Agreements (Intese Stato-Regioni)^2^ (art. 8, co. 6, Law n. 131/2003).

In this framework, a Legislative Decree (no. 56/2000) initiated a complex reform process aiming at introducing in the health care system principles of fiscal federalism, leading to a system of financing of the Regions based on their fiscal capacity and adjusted by equalizing measures.

This is a complex process that has remained unimplemented for a long time and has been relaunched in recent years (Legislative Decree 68/2011).

To date, health care is financed from different sources^3^:

- General taxation of the Regions;
- Co-participation of the special statute Regions and the Autonomous Provinces of Trento and Bolzano;
- Own revenues of National Health Service public healthcare institutions, e.g. tickets;
- The State budget, essentially through VAT and excise duties on fuel and through the National Health Fund (of which the main share is allocated to the Sicilian Region).^4^

The four sources take on a very different weight: in 2018 the general taxation of the Regions weighed 27%, the State budget 63%, of which 60% VAT, own revenues 2% and the co-participation of special statute Regions and Autonomous Provinces 8%.^5^

As part of this new framework, a monitoring system of the regional performance has been activated^6^ at the level of the State-Regions Conference.

Controls carried out since the early 2000s have revealed that strong regional differences still persist: they highlight the presence of regions with balanced budgets or with deficits that can be offset by the regions themselves (virtuous regions); and regions with high deficits that cannot be covered with ordinary budget measures, and important shortcomings in the LEAs provision (Ministero dell’economia e delle finanze, 2019).

For this reason, recovery plans—the so-called *Piani di Rientro*—have been introduced and have become a fundamental bilateral negotiation tools of the NHS governance system. Measures are identified for the re-balancing of the regional budget and of LEAs according to what is established at national level (Taroni, 2011). Unfortunately, these issues have not yet been solved, since seven Regions are still under recovery plans: Abruzzo, Calabria, Campania, Lazio, Molise, Puglia e Sicilia.^7^ Among these Calabria, and Molise are also put under the control of a Commissioner (Servizio Studi Camera dei Deputati, 2020).^8^

These considerations, despite their schematic nature, highlight the difficulties of the regionalisation process of the Italian National Health Service, started more than 25 years ago. The risk is that the regionalisation will not contribute to overcoming the regional imbalances that historically characterize Italy.

This process needs to be supported and deserves great attention from the academic world. The aim of this work is to contribute to understanding, through a system of metrics capable of measuring regional performance, whether the framework developed is actually sustainable for all regions, for the Italian State and ultimately for all Italian citizens.

### Conceptual Framework

The issue of performance measurement can be traced back to the 1980s when the reform of the public sector, according to the theory of the New Public Management (NPM), led to the introduction of performance measurement systems in the health sector (Nuti et al., 2017).

With NPM, the economic principles were introduced in the public sector with the aim of increasing efficiency and, in this respect, among other things, the public sector was asked to adopt more measurable and transparent performance standards.

Academics and international organizations developed conceptual frameworks and models to help countries build effective tools for performance analysis (Vainieri & Nuti, 2011). According to Lo Scalzo et al. (2009), in Italy the need for performance measurement has become increasingly evident since the early 1990s, when the reform of the National Health Service was taking place.

At institutional level, as already pointed out, monitoring activities are carried out annually to verify that the social insurance package (LEA) is provided by the Regions through a specific grid of indicators.^9^

Moreover, since 2008, the *Laboratorio Management e Sanità* (MeS Lab), in accordance with the National Agency for Health Services, has activated the Performance Evaluation System of Regional Health Systems (see Nuti et al., 2012). A process of inter-regional sharing has led to the selection of about 300 indicators aimed at describing and comparing, through a benchmarking process, the different dimensions of the performance of the health systems.^10^

The use of a large number of indicators leads to a precise and exhaustive description of the phenomenon in its components, but not to an overall view of the health system in its complexity. Starting from the observed variables, it is therefore necessary to extract latent concepts that can subdivide the phenomenon into several more easily measurable components. As argued by Jacobs et al., (2007, p. 384) “There is now a plethora of information available for the measurement of relative performance, and interpreting such data is therefore becoming increasingly complex. One widely adopted approach to summarizing the information contained in disparate indicators of health care performance is to create a single composite measure. The rationale for developing such a composite measure is that no single indicator can hope to capture the complexity of system performance”.

Within this framework, our main contribution is to evaluate the performance of the regional health care systems through composite indexes that aggregate several individual indicators into one single synthetic measure. In order to build composite indexes capable of capturing the different aspects of health performance we rely on the framework developed by the OECD in the HCQI project. Since the objective of the HCQI Project is to develop a set of indicators to question and compare the quality of health services in different OECD member countries (Mattke et al., 2006), we use this framework to build composite indexes to analyse the performance of Italian regions in its different dimensions.

It is worth noting that many scientists dispute the use of composite indexes to determine a single value for each geographical area, preferring the so-called dashboard, through which it is possible to identify various dimensions of the phenomenon, all relevant, without further aggregation. From a statistical point of view, this is an indisputable choice but, from a political point of view, single indexes could help the immediate understanding by the user. Indeed, the advantages of composite indexes are evident and can be summarized in: (1) a one-dimensional measurement of the phenomenon, (2) an easy interpretation compared to a batch of many single indicators and (3) a simplification of data analysis (Mazziotta & Pareto, 2013).

Within this framework, since it is widely recognized that health care services are multidimensional and that multiple aspects of performance require investigation(Jacobs et al., 2007), we propose a set of composite indexes reflecting the dimensions of the health care performance highlighted by the OECD HCQI framework as defined in 2015 by Carinci et al. (2015). Indeed, the framework has been developed since the 2000s in different stages.

The first framework by the OECD was presented by Arah et al. (2006) and was built on the basis of three key elements.

First, it was based on the American conceptual framework created by the Agency for Healthcare Research and Quality (AHRQ). The latter consists of a matrix that includes components of health quality (*e.g.* effectiveness, safety, timeliness, patient centrality, fairness) and patient needs (*e.g.* stay healthy, improve, and living with illness or disability, facing the end of life) (US Department of Health & Human Services, 2003).

The second pillar of the framework was the Canadian Health Indicator Framework (CHIF) developed by the Canadian Institute for Health Information (CIHI) and Statistics Canada. The CHIF was built following Lalonde (1974) and it has four main levels, namely health status (4 fields), non-medical determinants of health (4 fields), health system performance (8 dimensions or fields) and community and health system characteristics (3 fields) (Arah & Westert, 2005).

The third reference for the HCQI Project was the WHO and OECD proposals for the identification of social and economic goals of health policy (see Hurst & Jee-Hughes, 2001).

Combining these three models, the OECD framework presented in 2006 has four levels, each capturing a different aspect of health care performance.

The four tiers are: (1) “*Health”*, to capture in a broad sense the health of society; (2) the “*Non-health care determinants of health”*, such as lifestyles and socio-economic conditions; (3) the “*Health care system performance”*, to assess the process, inputs, and outcomes of the health care system; (4) and finally the “*Health system design and context”* to capture the characteristics of the general context and health system of a country.

**Fig. 1:**
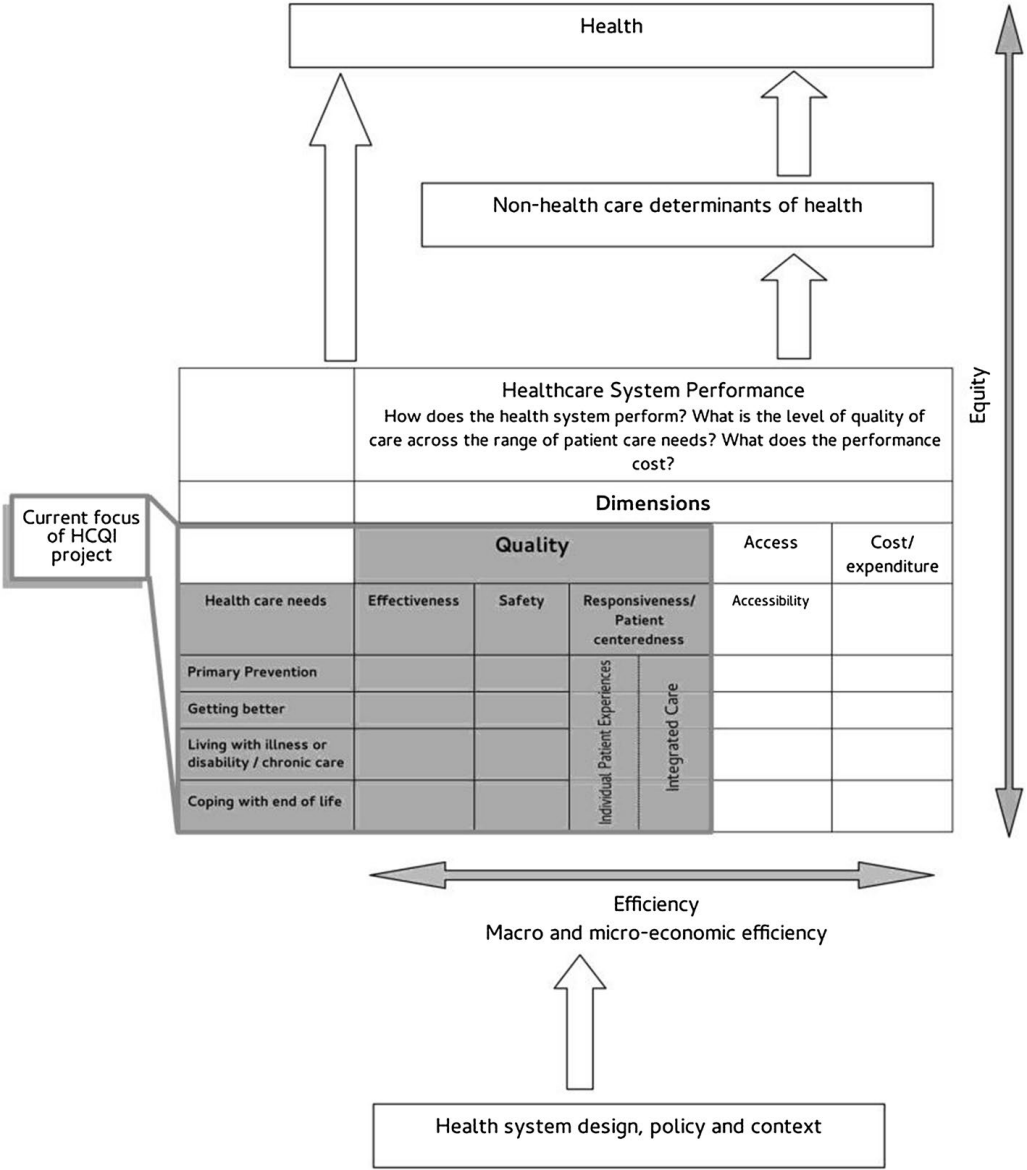
OECD Framework for performance measurement. Source: Carinci et al. (2015)

The framework was then revised and updated by Carinci et al. (2015). The main result is the provision of a new scheme for the allocation of indicators according to six different criteria, namely validity, reliability, relevance, actionability, international feasibility and international comparability (Carinci et al., 2015). Figure 1 shows the HCQI Project conceptual framework.

## Methodology and Data

To propose composite indexes of health system performance, we rely on a simplified version of the “*Healthcare System Performance*” tier (see Fig. 1), focusing only on health care performance dimensions without referring to healthcare needs (rows: primary prevention, getting better, living with illness or disabilities/chronic care, coping with the end of life). Nevertheless, the dimensions faithfully follow the OECD model: we construct three indexes for *Accessibility*, *Cost/Expenditure* and *Quality*. The latter is the result of the aggregation of three sub-indexes: *Effectiveness*, *Safety* and *Responsiveness/Patient Centeredness*.

Furthermore, for the sake of completeness, our analysis proposes two composite indexes which are intended to be representative of two other tiers of the OECD framework. Indeed, we build a *Health Status Index* and a *Lifestyles Index*, which respectively represent the first (“*Health”*) and second (“*Non-health care determinants of health”*) tier of the OECD framework.

### Data

Concerning the set of indicators we use to form the composite indexes, we refer to a sample consisting of 21 observations, *i.e.* the Italian Regions and the two Autonomous Provinces of Trento and Bolzano. The reference year of our analysis is 2015, with a few exceptions for which we use immediately previous or subsequent values. The main source of our indicators is the software "Health for all" (HFA), the territorial information system on health provided by ISTAT.^11^ Other sources are the annual reports provided by the National Health Observatory,^12^ the data provided by the "Passi" surveillance,^13^ and data from the SDO Report by the Italian Health Ministry (Ministero della Salute, 2019).

In order to select the indicators, we refer to both the list provided in Carinci et al. (2015) and in Vrijens et al. (2013)^14^ compatibly with the availability of data at NUTS-2 Italian level.

The indicators deemed suitable for the health care system performance indexes are shown in Table 1. In detail, since *Accessibility* describes if health services are easily reached, we include variables referring to household spending, cancer screening and the number of residential beds. The level of *Cost-Expenditure* is filled with three indicators, *i.e.* current public health expenditure per capita, the percentage of public expenditure on the total and the gross territorial pharmaceutical expenditure per capita charged to the NHS. Turning to *Quality*, variables are chosen respecting its components, *i.e. Effectiveness*, *Responsiveness /Patient-Centeredness* and *Safety*. *Effectiveness* is the degree to which the desired results are achieved, so the variables we include in the construction of this index are the 5-year net survival rate after diagnosis of neoplasia and the suicide mortality rate. The *Safety* dimension should capture the extent to which the system is able to prevent damage to the users, therefore it is calculated using the rate of sepsis-related mortality, the percentage of short hospital stays and the percentage of caesarean sections. The dimension of *Responsiveness / Patient-Centeredness*, according to Arah et al. (2006), is increasingly measured on the basis of patient experience and, therefore, the variables we use aim to capture the degree of patient satisfaction for hospital services.

**Table 1:** List of variable indicators used to construct the Healthcare System Performance Indexes by definition, source and year

Index	Indicators	Source	Year
Accessibility index	Family health expenditure (Eur)*	HFA	2014
	%Cervical cancer screening	PASSI	2013–2015
	%Breast cancer screening	PASSI	2010–2013
	No. residential social-health residential beds for the elderly and people with disabilities (for inhabitant)	Osserva Salute	2013
Qualtiy- effectiveness Index	5 years net survival rate after diagnosis of neoplasia (all locations except total skin)	PASSI	2015
	Suicide mortality rate, self-injury*	HFA	2015
Quality-safety index	% Caesarean sections*	SDO Report	2017
	Standardised rate (per 10,000 people) of sepsis-related mortality in the population of 75 + *	Osserva Salute	2016
	% Short hospital stays (2–3 days) out of total medical DRGs*	SDO Report	2017
Quality-patient centeredness index	%Very satisfied people for hospital medical care	HFA	2015
	%Very satisfied people in hospital toilets	HFA	2015
	%Very satisfied people hospital nursing care	HFA	2015
Cost/expenditure index	Current public health expenditure percapita	HFA	2013
	Public expenditure as a % of total expenditure	HFA	2013
	Gross territorial pharmaceutical expenditure per capita charged to the National Health Service	Osserva Salute	2012

The variables marked with an * are normalised in order to change their polarity from negative to positive

Turning to the *Health Status Index,* we include health status variables such as life expectancy and life expectancy in good health, a physical and psychological status index, and, finally, the infant mortality rate. To calculate the *Lifestyle Index,* we consider variables concerning obesity, smoking and eating habits, HIV infection. Table 2 shows in detail the list of indicators we consider eligible to compose the two indexes.

**Table 2:** List of variable indicators used to construct the Health Status and Lifestyle Index by definition, source and year

Index	Indicator	Source	Year
Health status index	Life expectancy	HFA	2015
	Healthy Life Expectancy	HFA	2013
	Infant Mortality rate*	HFA	2015
	Physical Status Index	HFA	2013
	Psychological Status Index	HFA	2013
Life style index	% Obese People 18 + *	HFA	2015
	% People Overweight 18 + *	HFA	2015
	% Smokers 15 + *	HFA	2015
	% Large Smokers 15 + *	HFA	2015
	Average no. of cigarettes daily 15 + *	HFA	2015
	% people eating an adequate breakfast 3 +	HFA	2015
	% persons not engaged in sport or physical activity 3 + *	HFA	2015
	%people consuming fruit or vegetables at least once a day 3 +	HFA	2015
	HIV rate*	HFA	2015
	% persons for whom the predominant drink is a hard drink*	HFA	2015

The variables marked with an * are normalised in order to change their polarity from negative to positive

Summing up, we construct and analyse the following indexes:

- Accessibility Index
- Quality Index, that is a multidimensional index resulting from the aggregation of three index: Effectiveness Index, Safety Index and Responsiveness/Patient Centeredness Index
- Cost-Expenditure Index
- Health Status Index
- Lifestyle Index

### Composite Indexes

Constructing a composite index is a complex task with phases that involve several alternatives and possibilities which affect the quality and reliability of the results. It is possible, shortly, to individuate the following steps to take (Mazziotta & Pareto, 2012): (1) defining the phenomenon to be measured (it should refer to a theoretical framework, linking various sub-groups and underlying indicators); (2) selecting a group of individual indicators chosen according to their relevance, analytical soundness, timeliness, accessibility, in order to minimize the redundancy (Salzman, 2003); (3) normalizing the individual indicators in order to make the indicators comparable; 4) aggregating the normalized indicators to combine all the components to form one or more composite indexes (Mazziotta & Pareto, 2013).

The theoretical framework to which we refer is analyzed in Sect. 2.2, while in Sect. 3.1, we presented the set of indicators we chose to compose the performance composite indexes.

The current section shows the methodology for normalizing the individual indicators and for aggregating the normalized indicators to form the composite indexes.

A fundamental issue, concerning composite index construction, is the degree of compensability or substitutability of the individual indicators. The components of a composite index are called ‘substitutable’ if a deficit in one component may be compensated by a surplus in another (e.g., a low value of “Life expectancy” can be offset by a high value of “Standardized mortality ratio” and vice versa). Similarly, the components of a composite index are called ‘non-substitutable’ if a compensation among them is not allowed (e.g., a low value of “Life expectancy” cannot be offset by a high value of “Physical status index” and vice versa). Therefore, we can define an aggregation approach as ‘compensatory’ or ‘non-compensatory’ depending on whether it permits compensability or not (Tarabusi & Guarini, 2013). In any composite index each dimension is introduced to represent a relevant aspect of the phenomenon considered, therefore a measure of unbalance among dimensions may help the overall understanding of the phenomenon. In a non-compensatory or partially compensatory approach, all the dimensions of the phenomenon must be balanced and an aggregation function that takes unbalance into account, in terms of penalization, is often used (Mazziotta & Pareto, 2017, 2019).

Due to the nature of the variables used, we opted for a non-compensatory approach: the Adjusted Mazziotta-Pareto Index (AMPI). The AMPI is a partially compensatory index that allows comparability of the data across units and over time (Mazziotta & Pareto, 2018). It is a variant of the Mazziotta-Pareto Index (MPI), based on a re-scaling of the individual indicators by a Min–Max transformation, in contrast with the classic MPI where all the indicators are normalized by a linear combination of z-scores (De Muro et al., 2011).

AMPI makes the indicators independent from the unit of measure. Therefore, all the individual indicators are assigned equal weights and absolute time comparisons are allowed (Mazziotta & Pareto, 2016). It is based on a non-linear function which, starting from the arithmetic mean, introduces a penalty for the units with unbalanced values of the indicators. Individual indicators are normalized by a re-scaling according to two ‘goalposts’, *i.e.* a minimum and a maximum value which represent the possible range of each variable for all time periods and for all units. Such type of normalization allows to perform absolute comparisons over time.

(Landi et al., 2018; Mazziotta & Pareto, 2016, 2018; Norman, 2010).

The steps for computing the Adjusted MPI are given below (Mazziotta & Pareto, 2018).

1. Normalization
  Given the matrix X = {xij} with n rows (units) and m columns (individual indicators), it is possible to calculate the normalized matrix R = {rij} as follow:
  1 $${r}_{ij}=\frac{{x}_{ij}-{Min}_{{x}_{j}}}{{Max}_{{x}_{j}}-{Min}_{{x}_{j}}}60+70$$
  where x_ij_ is the value of the indicator *j* for the unit *i* and ${Min}_{{x}_{j}}$ and ${Max}_{{x}_{j}}$ are the ‘goalposts’ for the indicator j.
  If the indicator j has negative ‘polarity’, the complement of (1) with respect to 200 is calculated. Indeed, the "polarity" of a single indicator is the sign of the relationship between the indicator and the phenomenon to be measured: if the single indicator represents a dimension considered positive the polarity is positive, while if it represents a dimension considered negative the polarity is negative. Since the phenomena we measure, *i.e.* health care system performance and health status/lifestyles, are all "positive", it is necessary to normalize the single indicators that have negative polarity.
2. Aggregation
  Denoting with ${M}_{{r}_{i}}$ and ${S}_{{r}_{i}}$, respectively, the mean and the standard deviation of the normalized values of the unit *i*, the generalized form of the AMPI is given by:
  2 $${AMPI}_{i}={M}_{{r}_{i}}-{S}_{{r}_{i} }\cdot {cv}_{i}$$
  where
  $${cv}_{i}=\frac{{S}_{{r}_{i} }}{{M}_{{r}_{i}}}$$
  is the coefficient of variation for the unit *i*.
  The AMPI decomposes the score of each unit in two parts: mean level (M_ri_) and penalty (S_ri_cv_i_). The penalty is a function of the indicators’ variability in relation to the mean value (‘horizontal variability’) and it is used to penalize the units. The aim is to reward the units that, mean being equal, have a greater balance among the indicators’ values. AMPI allows to compute the score of each unit independently of the others (Mazziotta & Pareto, 2019). The ‘price’ to pay for having scores comparable over time is that indicators with different variability are aggregated. However, normalized indicators in an identical range have much more similar variability than original ones (Mazziotta & Pareto, 2016).

## Results and Discussion

Following the HCQI framework, Sect. 2.2 identifies three phenomena to be observed at regional level, namely *heath care system performance*, *health status* and *lifestyles*, and Sect. 3.1 presents the indicators considered relevant to represent each one. All indicators have been normalised following Eq. 1 and then aggregated according to Eq. 2. Below, we present the calculated indexes and we discuss the comparison between Regions that can be derived from them. For each index we present the ranking of the Regions according to the index score.

In presenting the results, we start by analysing the *Health Status* and *Lifestyles Indexes* (Table 3), as we believe that they are a reference point for commenting on and interpreting the health system performance indexes.

**Table 3:** Health Status and Lifestyles Indexes

Region	Health status	Region	Lifestyles
Bolzano	127.73	Trento	115.56
Trento	118.97	Bolzano	111.77
Lombardia	108.65	Veneto	111.50
Friuli-Venezia Giulia	107.03	Friuli-Venezia Giulia	108.32
Veneto	106.37	Marche	107.25
Toscana	104.96	Valle d'Aosta	104.89
Emilia-Romagna	103.60	Lombardia	104.46
Liguria	102.84	Piemonte	103.24
Piemonte	100.90	Toscana	103.12
Lazio	100.41	Emilia-Romagna	100.56
Marche	97.51	Umbria	96.67
Abruzzo	97.41	Sardegna	96.31
Sardegna	94.83	Basilicata	95.74
Puglia	94.82	Abruzzo	93.56
Valle d'Aosta	92.00	Lazio	93.14
Umbria	90.64	Liguria	91.97
Molise	89.46	Puglia	89.69
Sicilia	87.62	Sicilia	89.01
Campania	86.58	Calabria	84.63
Calabria	84.65	Molise	83.31
Basilicata	82.76	Campania	80.87

The analysis of these two indexes shows a homogeneous picture and highlights a better performance for the Central-Northern Regions.

In particular, with regard to *Health Status Index*, we can observe the Autonomous Provinces of Bolzano (with an index of 127.73) and Trento (118.97) ranking in the first two positions, followed by Lombardia, Friuli Venezia Giulia and Veneto, with value rather similar and very far from the first two. In the last 5 positions we find Molise, Sicilia, Campania, Calabria and Basilicata, with values lower than 90.

Similarly, for the *Lifestyles Index*, the first two positions are occupied by the two Autonomous Provinces, even if in inverted positions. Following these, Veneto (111.50), Friuli Venezia Giulia (108.32) and Marche (107.25) are slightly detached. The last positions include Puglia, Sicilia, Calabria, Molise and Campania with values below 90.

As regarding the health care system performance indexes, Table 4 presents the results for *Accessibility*. Valle d'Aosta is in the first position with a value of 117.48, followed by the Autonomous Provinces of Trento and Bolzano, Friuli Venezia Giulia and Umbria. The last five places include Puglia (91.12), Lombardia (86.99), Sicilia (85.44), Campania (77.48) and Calabria which records a very low value (75.90).

**Table 4:** Accessibility index

Region	Accessibility
Valle d'Aosta	117.48
Trento	111.42
Bolzano	110.28
Friuli-Venezia Giulia	109.91
Umbria	107.25
Liguria	106.52
Marche	105.17
Toscana	102.84
Emilia-Romagna	102.84
Veneto	101.77
Molise	99.61
Piemonte	97.73
Lazio	97.29
Sardegna	94.97
Basilicata	93.45
Abruzzo	92.97
Puglia	91.12
Lombardia	86.99
Sicilia	85.44
Campania	77.48
Calabria	75.90

Valle d’Aosta owes this result primarily to the fact that it is the Region with the lowest household health expenditure and is the first Region for residential beds. With reference to the latter variable, the two Autonomous Provinces of Trento and Bolzano are in second and third place respectively, and the household expenditure is very low.

With reference to Lombardia, the high level of household spending stands out, which is very high compared to all the other Regions and therefore reduces the regional performance in terms of accessibility. In addition, it has a low percentage of residential beds. On the other hand, it has a high percentage of breast screening, second only to Emilia Romagna. As regards the last three Regions, Sicily, Campania and Calabria, they have very low values both in screening and in the percentage of residential beds. Finally, it should be noted that while Lazio occupies a central position in the general ranking, it has one of the lowest residential bed percentage values. Veneto and Emilia Romagna record high performance in terms of screening, but in terms of household health care expenditure, they follow Lombardy in second and third position.

The *Quality Index* (Table 5), divided into three sub-indexes *Effectiveness*, *Safety* and *Responsiveness*/*Patient-centeredness*, shows Lombardia in the first place (110.87), followed by Veneto (109.12), Trento (108.59), Toscana (105.88), Piemonte (104.19). The last 5 positions include Sardegna (87.64), Puglia (86.98), Calabria (83.99), Molise (80.83) and Campania (80.24).

**Table 5:** Quality index and its components (sub-indexes)

Region	Effectiveness	Safety	Responsiveness/Patient-centeredness	Quality
Lombardia	110.39	106.13	116.90	110.87
Veneto	114.31	110.61	103.35	109.14
Trento	94.13	108.13	135.07	108.59
Toscana	111.74	102.10	104.52	105.88
Piemonte	98.17	103.49	111.83	104.19
Abruzzo	94.05	103.65	111.36	102.29
Umbria	98.13	97.90	109.05	101.30
Emilia-Romagna	112.35	81.11	120.65	100.55
Friuli-Venezia Giulia	92.42	98.30	110.66	99.60
Valle d'Aosta	82.62	103.38	120.53	98.65
Liguria	102.14	96.78	96.11	98.23
Bolzano	89.73	92.25	121.55	98.09
Marche	86.18	102.13	102.39	96.01
Basilicata	92.84	101.49	90.07	94.42
Lazio	99.56	92.12	81.75	90.26
Sicilia	100.46	92.01	76.92	88.21
Sardegna	78.21	92.05	95.50	87.64
Puglia	104.59	96.23	70.55	86.98
Calabria	97.99	83.00	75.56	83.99
Molise	101.55	79.26	70.74	80.83
Campania	94.51	79.50	71.65	80.24

To understand the meaning of this index, it is necessary to observe it in its various components, *i.e.* each sub-index. With reference to *Effectiveness* (Column 1) the first three positions are occupied by Veneto, Emilia-Romagna and Tuscany. They have the highest values for the five-year survival rate after diagnosis of neoplasia, with Emilia-Romagna first, Tuscany second and Veneto third. The ranking of the *Effectiveness Index* is influenced by the suicide rate for which Veneto has a lower value than the other two Regions. These three Regions are followed by Lombardia, which holds the fourth position in both the *Effectiveness Index* and the 5-year survival indicator, and it shows a low value of the suicide rate. The last three positions of the Index are occupied by Marche, which is fourteenth for five-year survival rate and seventh for suicide rate, Valle d'Aosta, which is first for the latter indicator, and Sardinia, third for suicide rate and penultimate for five-year survival rate.

It is worth noting that many Northern Regions that record very high values in terms of *Health Status* (Table 3), such as Trento, Friuli Venezia Giulia and Bolzano, have low values in the *Effectiveness Index*. This result is mainly due to high suicide mortality rate, since these Regions are in fourth, fifth and second place respectively. For the five-year survival rate after diagnosis of neoplasia, instead, Bolzano and Trento are in a higher rank—sixth and seventh position respectively—than Friuli Venezia Giulia, which occupies the tenth place.

With reference to the *Safety* (Column 2), which includes both infections and variables related to organizational and clinical appropriateness, the first three positions for the *Safety Index* are held by Veneto, Trento and Lombardia. In detail, Veneto is the Region with the lowest number of short hospital stays out of total medical DRGs, immediately followed by Lombardia. Trento has the lowest percentage of caesarean sections, while Veneto and Lombardia are third and seventh in this indicator. Campania and Molise are in the penultimate and last position. Campania has the highest percentage of caesarean sections, followed by Molise, which in turn is also second for the indicator on short hospital stays. Finally, the very low value of the *Safety Index* of Emilia Romagna stands out. This result can be attributed to the fact that Emilia-Romagna is first for sepsis-related mortality rate in people over 75 years.

With reference to *Responsiveness/Patient Centeredness Index* (Column 3), measured in terms of patient satisfaction with respect to some hospital services, very high levels of satisfaction are highlighted for Trento, Bolzano, Emilia Romagna and Valle d'Aosta—which register values above 120—and very low levels in Sicilia, Calabria, Campania, Molise and Puglia. It is worth noting that, as far as this index is concerned, the rankings of its three component indicators are generally in line with the final ranking of the composite index and that this latter is the one that records the greatest gap between the maximum (135.07) and the minimum values registered (70.55).

The last index is that relating to *Cost/Expenditure* (Table 6), which includes three variables, two of which concern per capita expenditure. For this index, higher values are highlighted for Molise (119.2), Sardegna (117.72), Lazio (111.48), Puglia (106.84) and Campania (105.93), while Piemonte, Toscana, Friuli Venezia Giulia, Valle d’Aosta, Veneto and Emilia Romagna are the Regions that are at the lowest levels, recording values below 90.

**Table 6:** Cost/expenditure index

Region	Cost/expenditure
Molise	119.20
Sardegna	117.72
Lazio	111.48
Puglia	106.84
Campania	105.93
Sicilia	103.66
Abruzzo	101.58
Calabria	99.98
Liguria	99.60
Basilicata	98.93
Bolzano	98.47
Umbria	98.09
Marche	95.19
Lombardia	93.30
Trento	92.58
Piemonte	89.84
Toscana	88.71
Friuli-Venezia Giulia	88.05
Valle d'Aosta	86.66
Veneto	82.23
Emilia-Romagna	81.87

The ranking resulting from the index values shows a clear predominance of the regions of Southern Italy at the top of the ranking. Molise is the first Italian Region, being the Region with the highest percentage of public expenditure on total expenditure and the second Region for the indicator on public health expenditure per capita. In second and third place there are Sardinia and Lazio, which are respectively fifth and seventh for the latter indicator, fourth and sixth for the percentage of public expenditure and sixth and fifth for the per capita NHS pharmaceutical expenditure. The positions following the first three are also occupied by Southern Regions. While they are all penalized by low public health expenditure per capita, occupying the last positions, they are the first for NHS pharmaceutical expenditure and for the percentage of public expenditure on the total. Finally, the last positions are occupied, respectively, by Valle d'Aosta, Veneto and Emilia Romagna. The first, despite being the third Region for public expenditure per capita, is the last for the percentage of public expenditure on the total. Veneto, instead, is among the last five positions for all three indicators. Emilia Romagna is ninth in terms of public expenditure per capita but for the other two indicators it is among the last three positions.

Finally, we consider Spearman's correlation between indicators (Table 7). Indeed, previous literature recognises the importance of knowledge of the relationships between performance measures in health care especially for health policy makers (Cinaroglu & Baser, 2018). Healthcare is a very complex field where performance depends on different aspects and on how these aspects are related. In the light of this, we perform a correlation analysis which may lead to some interesting considerations on the relationship between different aspects of health and health system performance.

**Table 7:** Non-parametric spearman correlations

	Accessibility	Effectiveness	Safety	Responsiveness/Patient-centeredness	Quality	Cost/expenditure	Health status	Lifestyles
Accessibility	1.000	−.242	.334	.634**	.427	−.549**	.534*	.703**
Effectiveness	−.242	1.000	−.027	−.151	.271	−.149	.187	−.205
Safety	.334	−.027	1.000	.569**	.821**	−.573**	.488*	.699**
Responsiveness/patient-centeredness	.634**	−.151	.569**	1.000	.782**	−.732**	.704**	.816**
Quality	.427	.271	.821**	.782**	1.000	−.758**	.701**	.727**
Cost/expenditure	−.549**	−.149	−.573**	−.732**	−.758**	1.000	−.539*	−.751**
Health Status	.534*	.187	.488*	.704**	.701**	−.539*	1.000	.764**
Lifestyles	.703**	−.205	.699**	.816**	.727**	−.751**	.764**	1.000

*Correlation is significant at the 0.05 level (2-tailed)

**Correlation is significant at the 0.01 level (2-tailed)

In detail, *Health Status* and *Lifestyles* are highly correlated (0.764). Moreover, *Accessibility* shows correlation both with *Health Status* (0.534) and with *Lifestyles* (0.703), as well as *Quality*, correlated at 70% with the *Health Status Index* and at almost 73% with the *Lifestyles Index.* The only negative and significant correlations concern the *Cost Expenditure Index* and all the other indexes.

The strong correlation between the *Health Status Index* and the *Lifestyles Index* supports a well-established relation in the literature. Indeed, lifestyles can be considered as determinants of both physical and mental health. In particular, when analysing modern wealth societies, lifestyles play a strong role in determining diseases with high mortality and morbidity such as obesity, diabetes, and cancer (Walsh, 2011).

In this scenario, health status and lifestyle indices are two sides of the same coin and represent an important tool for policy makers. In fact, inequalities in lifestyle behaviours—such as physical activity and diet quality—reflect inequalities related to fields other than health, such as geography, race, but also income and education levels (Whitsel, 2017). This aspect has been clearly highlighted by the global COVID-19 pandemic. Indeed, mortality from COVID-19 has a clear social gradient, which reaffirms the importance of the social determinants of health (OECD, 2020), which in turn are reflected in people's lifestyles.

Turning to the health care system performance indexes, the mixed evidence on the correlation analysis leads to a first general consideration. As we have seen, considering the different tiers of performance separately is necessary from a conceptual point of view, and our results underline this need: it would be misleading to consider these dimensions in a single multidimensional index since it would lead to an overly forced simplification of the complexity of the phenomenon under scrutiny. While composite indices allow the immediacy of information, one should not fall into the trap of over-aggregating data. In the context of health performance, for example, we argue that each of the composite index we computed represents an aspect of performance that deserves separate in-depth analysis, with no need to construct a single performance index.

A number of interesting considerations can be also made when looking at the individual performance indices in detail.

First, observing the indices that compose the *Quality Index*, that is *Safety, Effectiveness,* and *Responsiveness/Patient-Centeredness*, although they contribute to determine a single dimension, they are not always correlated with each other. This reflects the fact that Quality in the health system is a multi-faceted concept that requires careful analysis by researchers. Literature argues that exploring healthcare quality is methodologically difficult. Furthermore, it is important to remember that quality of health care is also defined differently according to the perspective adopted: different stakeholders have their own interests and definitions of quality, based on the importance they give to different elements of health services (Mosadeghrad, 2013).

Second, our *Accessibility Index* shows positive correlation with health status, thus confirming those literature that recognizes accessibility as an important facilitator of people' s overall health (Guagliardo, 2004).

Finally, the fact that the expenditure index is negatively correlated with other health care system performance indexes and also with *Health Status Index* and *Lifestyles Index* deserves further consideration. We attribute this result mainly to the fact that higher expenditure does not necessarily lead to an improvement in health status. This is linked to the concept of efficiency, i.e. the efficiency of transforming expenditure into better health outcomes (Ray & Linden, 2020). The relationship between health care quality and costs has been largely investigated in the literature and plays an important role in the political debate (Hussey et al, 2013; Weinstein & Skinner, 2010).

## Conclusion

In this article we investigated the performance of the regional health care systems in Italy. Since the 90’s the National Healthcare Service has been the subject of an important reform of regionalisation that assigned to the Regions the responsibility to provide health services and to guarantee regional economic sustainability. In Italy—a country historically characterized by a strong North–South economic and social dualism still persisting in the 1990s—the main risk recognised at the time was that regionalisation could lead to different regional systems and so accentuate disparities among Regions, in contrast to the Italian Constitution principles.

For our investigation, we constructed a set of composite indexes to investigate whether the regionalisation process of the Italian healthcare system is contributing to reduce or increase regional disparities. For the construction of the indexes and for the choice of the dimensions to be evaluated, we drew inspiration from the framework developed by the OECD in the Health Care Quality Indicator project. The objective of the HCQI Project is to develop a set of indicators to investigate the quality of health services in different OECD member countries and to investigate the differences between countries; for this reason, we consider this framework well suitable for our comparison.

Our results highlight that regional disparities persist between the Southern and Northern Regions, consistently with the evidence from both institutions and the academic world. In Italy, there are both Regions with good performance and Regions with serious deficits that also show shortcomings in the provision of LEAs. According to our analysis, most of the Southern Regions have worse performance levels in terms of both health services provision and health status and lifestyles of the population.

We are aware that our methodology and dataset have some limits that must be overcome by further research. The number of indicators used to calculate composite indexes should be increased. Moreover, further analysis concerning the valid application of the indicators in Italy is needed. We refer in particular to the *Cost/Expenditure Index*. A high level of public health expenditure can be considered a proxy for inclusion and cohesion, but, as highlighted in Sect. 2.1, one of the objectives of the reform of the Italian health system, which is based on solidarity principles, is the rationalisation of expenditure. Our *Cost/Expenditure Index*, however, captures only the first of these two aspects. Therefore, it should be integrated with indicators that also capture efficiency and economic sustainability.

Finally, it is worth noting that the COVID-19 pandemic has highlighted the need to consider health system resilience as an equally important dimension of health system performance alongside accessibility, quality of care and efficiency (OECD, 2020), showing the relevance of each single dimension we consider in this study but also the importance of continuous refinement of the dimensions considered in assessing health performance.

Continuous research is still needed to provide national and regional policy-makers with data and tools to find the most appropriate institutional framework and measures to encourage a cohesive and sustainable Italian health system to the benefit of the entire population.

## Appendix: Regional Health Care Funding in Italy

Health care funding sources:

- General taxation of the Regions: regional tax on productive activities—IRAP (in the revenue component allocated to the financing of health care), and regional additional component to personal income tax (IRPEF), calculated on national base rates, not taking into account any increased revenue from regional tax manoeuvres that may be activated to face deficits;
- Co-participation of the Regions with special statute and the Autonomous Provinces of Trento and Bolzano, which participate in health financing up to the amount of the needs not met by the sources described in the previous points, except for the Sicilian Region, for which the co-participation rate has been set since 2009 at 49.11% of its financing (Law 296/2006 art. 1, paragraph 830);
- Own revenues of National Health Service public healthcare institutions (*i.e. Aziende sanitarie locali* and hospitals) (tickets and revenues from the intramoenia activity);
- The State budget: it finances what is not covered by other sources of financing, essentially through VAT and excise duties on fuel and through the National Health Fund (of which the main share is allocated to the Sicilian Region).^15^

See Tables 8 and 9.

**Table 8:** NHS funds allocated to Regions, Tab. C, 2018, values in Euros*.* Source: Comitato Interministeriale per la programmazione economica, Delibera 28 novembre 2018, Fondo sanitario nazionale 2018—Riparto delle disponibilità finanziarie per il Servizio sanitario nazionale (Delibera n. 72/2018, OJ Serie Generale n.49 27–02-2019)

Regions and Autonomous Procinces	Own revenues of the National Health Service entities	Co-participation of the Regions with special statute and the Autonomous Provinces	IRAP	Additional IRPEF	State Budget		Total (before mobility)
					Integration Dlgs. 56/2000 (VAT)	National Health Fund	
	(1)	(2)	(3)	(4)	(5)	(6)	(7) = (1) + (2) + (3) + (4) + (5) + (6)
PIEMONTE	167.095.971		1.533.600.000	762.053.000	5.650.623.450	21.247.752	8.134.620.173
VALLE D'AOSTA	4.341.336	135.787.600	68.550.000	23.267.000			231.945.936
LOMBARDIA	344.688.926		4.932.950.000	1.889.244.000	10.943.133.997	47.427.521	18.157.444.444
P.A. BOLZANO	17.089.038	464.720.424	344.950.000	101.023.000			927.782.462
P.A.TRENTO	17.328.157	585.068.631	274.250.000	92.676.000			969.322.788
VENETO	187.978.900		1.974.000.000	814.614.000	5.912.745.465	23.279.894	8.912.618.259
FRIULI VENEZIA GIULIA	47.484.584	1.459.051.250	543.700.000	216.519.000			2.266.754.834
LIGURIA	62.729.872		502.100.000	281.047.000	2.118.430.826	7.763.094	2.972.070.792
EMILIA ROMAGNA	171.955.829		1.903.700.000	810.204.000	5.256.844.641	21.324.568	8.164.029.038
TOSCANA	138.369.096		1.390.250.000	617.674.000	4.767.474.777	18.106.160	6.931.874.033
UMBRIA	34.031.402		209.850.000	131.401.000	1.264.764.509	4.295.046	1.644.341.957
MARCHE	57.467.177		463.950.000	226.810.000	2.076.218.835	7.396.817	2.831.842.829
LAZIO	162.193.247		2.598.900.000	954.120.000	6.879.741.925	27.746.659	10.622.701.831
ABRUZZO	41.537.068		290.050.000	167.416.000	1.912.186.005	6.314.556	2.417.503.629
MOLISE	12.952.736		10.000.000	34.354.000	511.922.440	1.490.729	570.719.905
CAMPANIA	163.215.831		964.350.000	547.172.000	8.528.339.728	26.720.388	10.229.797.947
PUGLIA	113.350.898		651.600.000	418.720.000	6.092.924.078	19.056.352	7.295.651.328
BASILICATA	16.926.354		43.400.000	60.657.000	912.029.101	2.705.311	1.035.717.766
CALABRIA	47.418.994		27.400.000	176.378.000	3.261.262.264	9.198.624	3.521.657.882
SICILIA	128.084.893	4.430.893.309	1.199.350.000	478.229.000		2.785.827.870	9.022.385.072
SARDEGNA	45.917.138	2.238.563.864	535.250.000	196.335.000			3.016.066.002
TOTAL	1.982.157.447	9.314.085.078	20.462.150.000	8.999.913.000	66.088.642.041	3.029.901.341	109.876.848.907

**Table 9:** NHS funds allocated to Regions, Tab. C, 2018, values %*.* Source: our elaboration from Comitato Interministeriale per la programmazione economica, Delibera 28 novembre 2018, Fondo sanitario nazionale 2018—Riparto delle disponibilità finanziarie per il Servizio sanitario nazionale (Delibera n. 72/2018, OJ Serie Generale n. 49 27–02-2019)

REGIONS AND AUTONOMOUS PROCINCES	Own revenues of the National Health Service entities (%)	Co-participation of the Regions with special statute and the Autonomous Provinces (%)	IRAP (%)	Additional IRPEF (%)	State Budget		Total (before mobility (%)
					Integration Dlgs. 56/2000 (VAT) (%)	National Health Fund (%)	
	(1)	(2)	(3)	(4)	(5)	(6)	(7) = (1) + (2) + (3) + (4) + (5) + (6)
PIEMONTE	2	0	19	9	69	0	100
VALLE D'AOSTA	2	59	30	10	0	0	100
LOMBARDIA	2	0	27	10	60	0	100
P.A. BOLZANO	2	50	37	11	0	0	100
P.A.TRENTO	2	60	28	10	0	0	100
VENETO	2	0	22	9	66	0	100
FRIULI VENEZIA GIULIA	2	64	24	10	0	0	100
LIGURIA	2	0	17	9	71	0	100
EMILIA ROMAGNA	2	0	23	10	64	0	100
TOSCANA	2	0	20	9	69	0	100
UMBRIA	2	0	13	8	77	0	100
MARCHE	2	0	16	8	73	0	100
LAZIO	2	0	24	9	65	0	100
ABRUZZO	2	0	12	7	79	0	100
MOLISE	2	0	2	6	90	0	100
CAMPANIA	2	0	9	5	83	0	100
PUGLIA	2	0	9	6	84	0	100
BASILICATA	2	0	4	6	88	0	100
CALABRIA	1	0	1	5	93	0	100
SICILIA	1	49	13	5	0	31	100
SARDEGNA	2	74	18	7	0	0	100
TOTALE	2	8	19	8	60	3	100

## Footnotes

1 The LEAs were defined by the Agreement dated 8 August 2001, approved by the Prime Minister’s Decree on 29 November 2001. On 12 January 2017 the Prime Minister’s Decree updated the LEAs defined in 2001.

2 The Permanent Conference for relations between State, Regions and Autonomous Provinces of Trento and Bolzano is the institution called upon to foster cooperation between State, Regions and Autonomous Provinces, where political negotiations between the central government and the Regions take place. The new governance model also provides for the Conference of Regions and Autonomous Provinces, where decisions and actions taken at regional and province level can be coordinated.

3 For a complete description of the sources see Appendix and Tables 8 and 9 where 2018 values are presented.

4 See website Ministero della Salute, *Il finanziamento del Servizio Sanitario Nazionale*, available at: http://www.salute.gov.it/portale/temi/p2_6.jsp?lingua=italiano&id=4752&area=programmazioneSanitariaLea&menu=dati, (consulted on 5th December 2019).

5 With reference to the most important item, VAT, it is worth underlining that Legislative Decree 56/2000 provides that the annual quota pertaining to each Region is determined on the basis of the households’ average final consumption measured regionally by Istat for the previous three years. In the event that the VAT needed by a Region is lower than the VAT due on the basis of per capita consumption, the deficit Regions can draw on a special fund, which is fed by the Regions with VAT surplus (Camera dei Deputati, 2018). In 2017 this fund amounted to € 5.36 billion and 43% of it was fed by Lombardia, 16% by Lazio, 15% by Emilia Romagna, 9% by Veneto and Piemonte, 5% by Toscana and 3% by Liguria (Conferenza delle Regioni e delle Provincie Autonome, 2019).

6 In 2005, two Monitoring Tables were set up: the Table for the verification of regional requirements, coordinated by a representative of the State Accounting Department (Dipartimento della Ragioneria generale dello Stato), and the Standing Committee for the LEA provision. The State, the Regions, the National Agency for Regional Health Services (AGENAS) and the Italian Drugs Agency (AIFA) participate in both Tables.

7 Liguria and Sardegna concluded their recovery plans at the end of the first three-year period 2007–2009; Piemonte at the end of the 2013–2015 three-year period (Servizio Studi Camera dei Deputati, 2019).

8 The commissioning procedure is started by the State in the Regions where the Monitoring Tables, during the periodic checks, highlight non-compliance in relation to the financial balances. The Commissioner must adopt all the measures indicated in the recovery plan, as well as any further regulatory, administrative, organizational and managerial act for the complete implementation of the recovery plan (http://www.salute.gov.it/).

9 According to the State-Regions agreement of 23 March 2005, this verification allows the regions involved (excluding: Valle d'Aosta, the two Autonomous Provinces of Bolzano and Trento, Friuli Venezia Giulia and Sardinia since 2010) to have access to a reward mechanism (Ministero della Salute Direzione Generale della Programmazione Sanitaria – Ufficio VI, 2019).

10 For further details see http://performance.sssup.it/netval/start.php.

11 Notice that in our work we have limited problems of comparability between indicators because our analysis is carried out on Regions belonging to the same country.

12 The *Osservasalute Report* provides annually data and indicators with a critical analysis of the health status of Italians and the quality of health care at regional level. For further details see url: www.osservatoriosullasalute.it/.

13 “Passi” Surveillance has been launched in 2006 with the aim of carrying out a deep monitoring of the health status of the Italian adult population. For further detail see url: https://www.epicentro.iss.it/passi/.

14 In addition to international frameworks, countries such as the Netherlands and Belgium also adopted methodological frameworks to measure the performance of their national health care systems. More precisely, the Netherlands has been one of the first countries in the world to develop a national health care system performance network (van den Berg et al., 2014), while the Belgian framework has been constructed on the basis of the Dutch and the Canadian ones (Vrijens et al., 2013).
